# Autophagy mediates SUMO-induced degradation of a polyglutamine protein ataxin-3

**DOI:** 10.1080/19768354.2017.1330765

**Published:** 2017-05-25

**Authors:** Soo Pyung Hwang, Do Hee Lee

**Affiliations:** Department of Bio and Environmental Technology, College of Natural Sciences, Seoul Women’s University, Seoul, South Korea

**Keywords:** Ataxin-3, SUMO, autophagy, SUMO-interacting motif

## Abstract

Previously, we reported that small ubiquitin-like modifier-1 (SUMO-1) promotes the degradation of a polyglutamine (polyQ) protein ataxin-3 and proposed that proteasomes mediate the proteolysis. Here, we present evidence that autophagy is also responsible for SUMO-induced degradation of this polyQ protein. The autophagy inhibitor 3-MA increased the steady-state level of ataxin-3 and stabilized SUMO-modified ataxin-3 more prominently than the proteasome inhibitor MG132. Interestingly, SUMO-1 overexpression enhanced the co-localization of ataxin-3 and autophagy marker LC3 without increasing LC3 puncta formation suggesting that SUMO-1 is involved in the substrate recruitment rather than the induction of autophagy. To assess the importance of a putative SUMO-interacting motif (SIM) in ataxin-3 for SUMO-induced degradation, we constructed a SIM mutant of ataxin-3. Substitution of putative SIM (V165G) facilitated the degradation of polyQ-expanded ataxin-3, which is more resistant to SUMO-induced degradation than the normal ataxin-3. These results together indicate that SUMO-1 promotes the degradation of ataxin-3 via autophagy and the putative SIM of ataxin-3 plays a role in this process.

## Introduction

Expansion of trinucleotide CAG tract is the cause for the inherited neurodegenerative disorders collectively known as polyglutamine (polyQ) diseases. Expansion of polyQ repeats within the disease proteins is assumed to confer toxic gain-of-function and promote the formation of aggregates containing polyQ proteins (Durcan & Fon 2013). Ataxin-3, whose mutated form causes spinocerebellar ataxia type 3 (SCA3; also known as Machado–Joseph disease), is a polyQ protein with deubiquitinating function (Nascimento-Ferreira et al. 2011). Although the expansion of polyQ tracts presumably leads to inefficient degradation of disease proteins and increases their propensity to form large aggregates inside the cells, it is still debatable whether the large insoluble inclusions are cytotoxic or cytoprotective (Todd & Lim 2013).

Like ubiquitin (Ub), small Ub-like modifiers (SUMO) are reversibly conjugated to specific lysine residues in target proteins and affect protein–protein interaction, protein stability and subcellular localization (Sarge & Park-Sarge 2009). SUMOylation is implicated in the pathogenesis of a number of human diseases including cancer, cardiovascular and neurodegenerative diseases (Dorval & Fraser 2007; Bettermann et al. 2012). Involvement of SUMO proteins in polyQ diseases has also been reported. In the brain of SCA7 patients, SUMO-1 and SUMO-2 are co-localized with ataxin-7 aggregates and SUMOylation decreases the aggregation propensity as well as the toxicity of polyQ-expanded ataxin-7 (Janer et al. 2010). SUMOylation of ataxin-1 and the formation of intracellular aggregates containing ataxin-1 were promoted by oxidative stress (Ryu et al. 2010). By contrast, SUMO-1 overexpression facilitates the degradation of ataxin-3 (Jung & Lee 2013).

Although SUMO and Ub have distinct functions in proteostasis, recent findings indicate that SUMO plays a role in the regulated proteolysis. Large-scale mapping revealed that a quarter of SUMO acceptor lysines in the endogenous proteins can also be modified by Ub indicating that SUMOylation has a regulatory role in protein degradation by Ub-proteasome system (UPS) (Hendriks et al. 2014). Furthermore, a novel class of Ub ligases termed SUMO-targeted Ub ligases (STUbL) that recognize protein-conjugated SUMO and catalyze the formation of SUMO-Ub hybrid chain has been identified (Sriramachandran & Dohmen 2014). Recently, the interplay between SUMOylation and autophagy–lysosome system has also been proposed. Overexpression of Ubc9 (E2 enzyme for SUMO) increases autophagic flux in cardiomyocytes and reduce aggregate formation in autophagy-impaired transgenic mice (Gupta et al. 2016). These observations raise a possibility that SUMO functions in the degradation of neurodegenerative proteins via both autophagy–lysosome system and UPS.

To understand if ataxin-3 is also degraded by the autophagy–lysosome system, we compared the effects of selective inhibitors of UPS and autophagy on the SUMO-induced degradation of ataxin-3. In addition, we examined the effects of SUMO-1 overexpression on the co-localization of autophagic marker LC3 and ataxin-3. Finally, we investigated the role of a putative SUMO-interacting motif (SIM) in the SUMO-induced degradation of ataxin-3.

## Material and methods

### Plasmids, cell culture and DNA transfection

HA-tagged ataxin-3 (26Q and 73Q) and the SIM mutant (V165G) of ataxin-3 on pcDNA3.1/zeo plasmids were generated in the laboratory. Expression plasmids for FLAG-tagged SUMO-1 and myc-tagged Ub were provided by Prof. Chin Ha Chung (Seoul National University). BOSC 23 cells, a derivative of HEK 293T cells, were maintained in Dulbecco's modified Eagle medium/10% fetal bovine serum medium supplemented with L-glutamine and antibiotics at 37°C with 5% CO_2_. Plasmids were transfected into cells using Lipofectamine™ 2000 (Invitrogen). Unless otherwise specified, 1 µg of plasmid was used for transfection.

### Immunoblot assay

After 48 h of transfection with ataxin-3 and SUMO-1, cells were incubated with 5 µM MG132 or 5 mM 3-MA (methyladenine) for 24 h. Cells were then collected and boiled for 5 min with 1× SDS sample buffer at 95°C. Extracted proteins were separated by 8–10% SDS-polyacrylamide gel electrophoresis (SDS-PAGE) and then transferred to the Polyvinylidene fluoride membrane. After incubating with an anti-HA antibody (1: 1000, Sigma) or anti-β-actin antibody (1:1000, Santa Cruz) for 2 h, the membranes were incubated with horseradish peroxidase-conjugated anti-mouse IgG (1: 2000, Sigma) for 1 h. The proteins were visualized by using an enhanced ECL detection kit (ATTO) and images were obtained by using a chemiluminescent image analyzer (Fuji).

### Ubiquitylation assay

After transfection with ataxin-3, SUMO-1 and Ub (when necessary, 5 mM MG132 was added), cells were collected and lysed in NP-40 lysis buffer (20 mM Tris-HCl, pH 7.5; 150 mM NaCl; 1% NP-40; 1% glycerol) supplemented with protease inhibitors (Complete-MINI from Roche) and 5 mM *N*-ethylmaleimide to block proteolytic activities including SUMO proteases. After incubating 30 min on ice, the cell lysate was clarified by centrifugation at 1000 × *g* for 10 min. To isolate ataxin-3 proteins, 400 µg of cell lysate was mixed with 20 µl of anti-HA-agarose beads (Sigma) at 4°C for overnight. The protein complexes were collected by centrifugation, washed with phosphate-buffered saline with Triton-X-100 and then boiled in 1× SDS sample buffer. Ubiquitylated ataxin-3 was detected by immunoblot with anti-myc antibody (1:1000, Cell Signaling).

### Immunocytochemistry

To measure the co-localization of ataxin-3 and LC3 signals, BOSC 23 cells grown on cover glasses were transfected with ataxin-3 and SUMO-1. After 24 h, cells were washed with PBS, fixed with the 3.5% paraformaldehyde and then permeabilized with 0.1% triton X-100 for 15 min at room temperature. After incubation with an anti-HA antibody (1:200, Sigma) and anti-LC3 antibody (1:200, MBL) in 1% bovine serum albumin (BSA) in phosphate buffered saline (PBS) at room temperature for 2 h, cells were washed with PBS and then incubated with FITC-conjugated goat anti-mouse antibody (for ataxin-3) and Cy3-conjugated goat anti-rabbit antibody (for LC3) (1:500, Bethyl) in 1% BSA/PBS for 1 h. Finally, cells were incubated with 4′,6-diamidino-2-phenylindole (DAPI) for 15 min at room temperature for nuclear staining. Images were obtained using a laser confocal fluorescence microscope (C1+, Nikon).

### Colocalization analysis

To quantify the colocalized signals of ataxin-3 and LC3, confocal images (three independent images were selected from each group) were analyzed by ImageJ (using Coloc 2 plugin; http://imagej.net/Coloc_2) and the pixel intensity correlation (Pearson correlation coefficient *r*) was determined.

### Chase experiment

To determine the effect of SUMO-1 on ataxin-3 degradation, BOSC cells expressing ataxin-3 (normal and SIM mutant) and SUMO-1 were treated with cycloheximide (20 µg/ml) to block protein synthesis. From this point (*t* = 0), cells were collected every 8 h (for additional 24 h) and then processed for immunoblot with an anti-HA antibody.

## Results

### Proteolytic pathways mediating SUMO-induced ataxin-3 degradation

**Figure 1.**
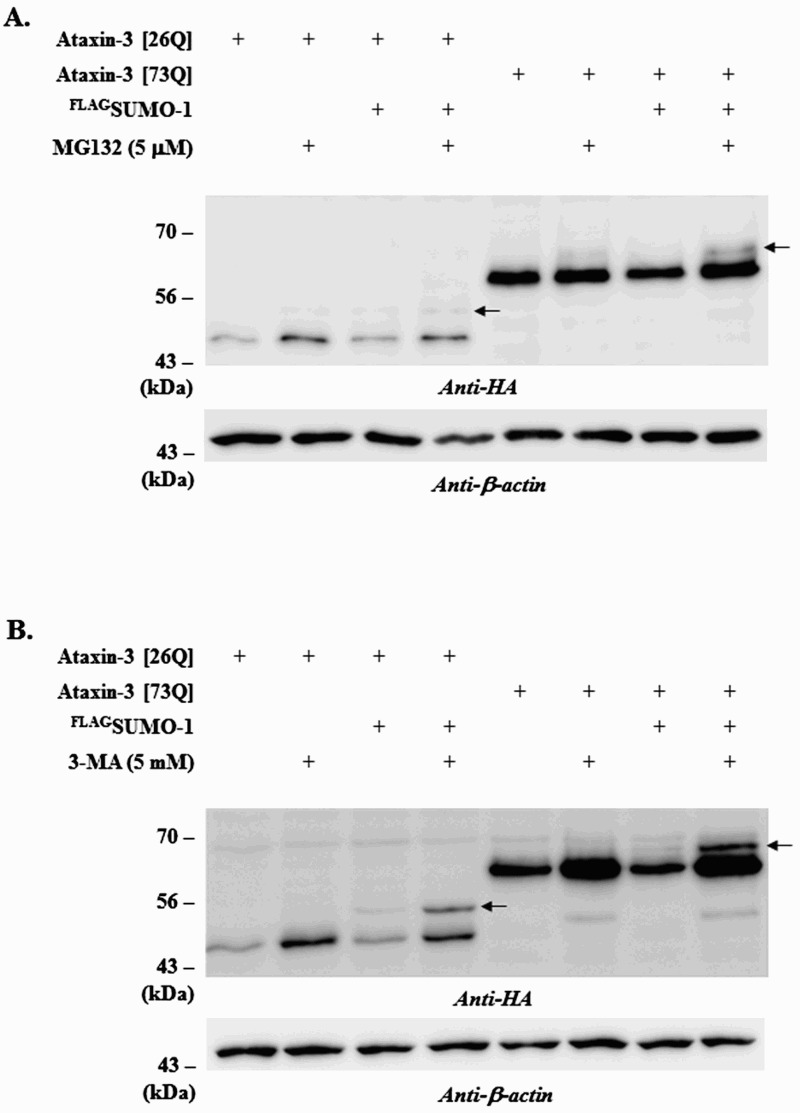
Effects of MG132 or 3-MA treatment on ataxin-3 degradation. To assess the involvement of different proteolytic pathways on ataxin-3 degradation, BOSC cells expressing HA-tagged ataxin-3 (26Q or 73Q) and FLAG-tagged SUMO-1 were incubated with 5 µM MG132 (a proteasome inhibitor) **(**A**)** or 5 mM 3-MA (an autophagy inhibitor) **(**B**)**. After 24 h, cells were collected, lysed and the cell lysate was subjected to immunoblot analysis using anti-HA antibody (ataxin-3) and anti-β-actin antibody. Arrows indicate the SUMO-modified form of ataxin-3.

Using a conventional immunoprecipitation method, we could not detect SUMO-modified form of ataxin-3 (Jung & Lee 2013). However, a similar study demonstrated the presence of the SUMOylated ataxin-3 by employing affinity isolation and TCA precipitation (Zhou et al. 2013). A plausible explanation for this discrepancy is that SUMOylated ataxin-3 is susceptible to proteolysis and thus difficult to detect unless proteolytic activities are compromised. To prove this possibility, we utilized the selective inhibitors of UPS and autophagy and examined their effects on the steady-state levels of ataxin-3 as well as SUMO modification. Proteasome inhibitor MG132 increased the steady-state levels of ataxin-3 (both normal [26Q] and polyQ-expanded [73Q] forms) to a certain extent. In addition, a slow-migrating band (indicated by arrows), which may represent the SUMOylated ataxin-3, was detected in the MG132-treated cells although they were only barely visible (Figure 1(A)). Interestingly, the autophagy inhibitor 3-MA reduced SUMO-induced degradation of ataxin-3 much more effectively. The slow-migrating form of ataxin-3 (indicated by arrows) was also more prominent in the cells treated with 3-MA (Figure 1 (B)). We tried again to detect SUMOylated ataxin-3 in the presence of 3-MA; however, the attempts were still unsuccessful (data not shown) suggesting that only a small fraction of ataxin-3 proteins may be SUMOylated. Nonetheless, these results clearly demonstrated that SUMO-induced degradation of ataxin-3 is mediated by autophagy.

**Figure 2.**
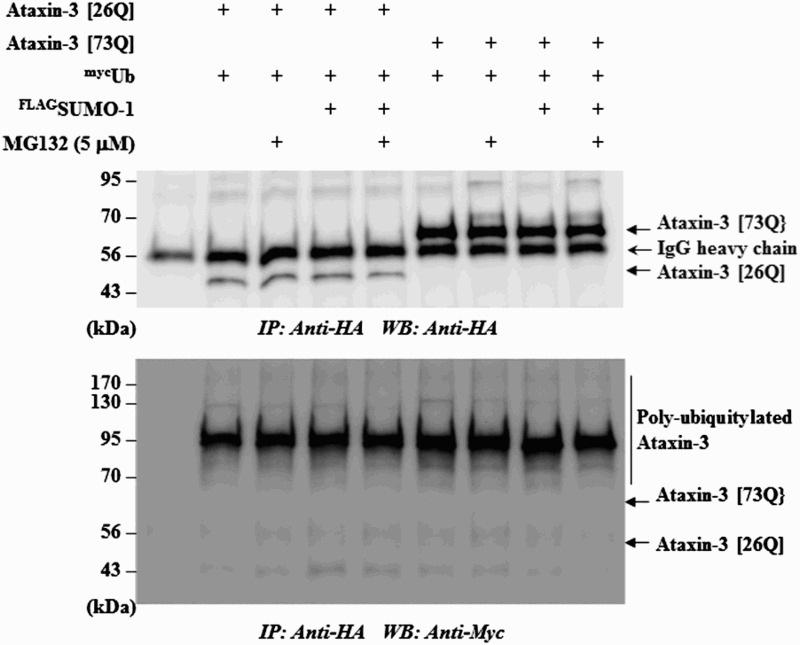
SUMO-1 overexpression does not increase ubiquitylation of ataxin-3. To test if SUMO-1 overexpression leads to the increased ubiquitylation of ataxin-3, HA-tagged ataxin-3 (26Q or 73Q), myc-tagged ubiquitin (Ub) and FLAG-tagged SUMO-1 were transfected into BOSC cells. When necessary, cells were incubated with MG132 (5 µM) to block the degradation of ataxin-3. The cell lysate was prepared and subjected to immunoprecipitation with anti-HA agarose beads followed by immunoblot with anti-HA antibody to ensure that ataxin-3 proteins were properly isolated (upper panel) or with anti-myc antibody to detect poly-ubiquitylated ataxin-3 (bottom panel).

On the other hand, the observation that MG132 blocked SUMO-induced degradation of ataxin-3, although the effect was not strong, suggested a role of UPS for this process. For example, STUbL proteins mediate the poly-ubiquitylation of SUMO-conjugated substrates and target them for destruction (Geoffroy et al. 2010; Her et al. 2015). To determine whether SUMO-1 also exerts similar effects, we examined the ubiquitylation of ataxin-3. As shown in Figure 2, SUMO-1 did not further increase the ubiquitylation of ataxin-3 even in the presence of MG132. These observations are in accordance with the previous report showing that the mutation of SUMO acceptor (K166R) did not affect ubiquitylation of ataxin-3 (Zhou et al. 2013). These findings, together with the effects of the autophagy inhibitor 3-MA, led us to speculate as to whether autophagy–lysosome system plays more important role in SUMO-induced ataxin-3 degradation.

### Autophagy and SUMO-induced ataxin-3 degradation

**Figure 3.**
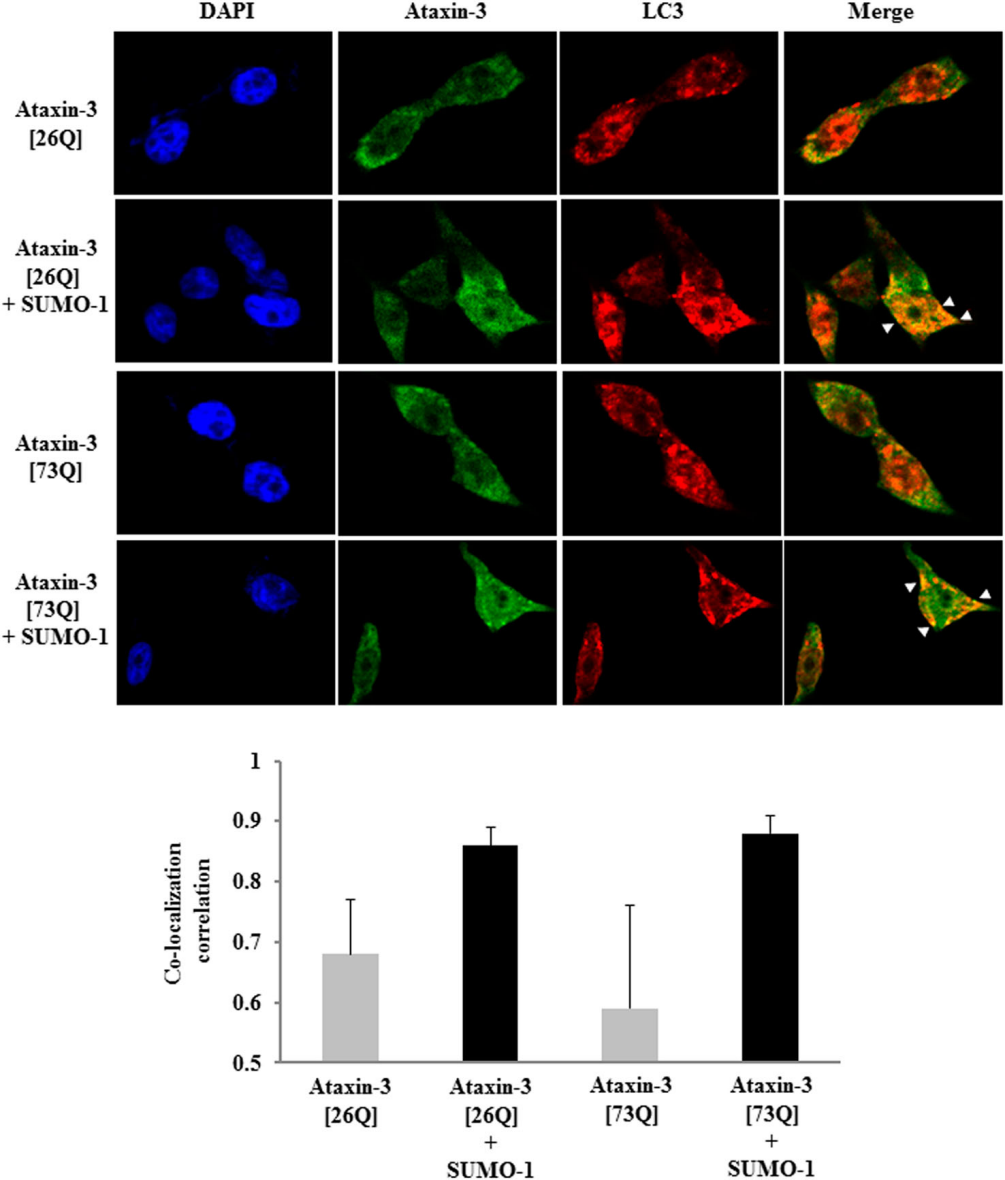
SUMO-1 enhances the co-localization of ataxin-3 and LC3. After 24 h of transfection of HA-tagged ataxin-3 (26Q or 73Q) with FLAG-tagged SUMO-1, BOSC cells were fixed and permeabilized. To determine the localization of ataxin-3 and LC3 (autophagy marker), cells were incubated with anti-HA antibody followed by FITC-anti-IgG antibody (for ataxin-3) and anti-LC3 antibody followed by Cy3-anti-IgG antibody (for LC3). DAPI was used for nuclear staining. Arrowheads indicate co-localization of ataxin-3 and LC3 (upper panel). For quantification of signal co-localization, images were analyzed by ImageJ (Coloc 2 plug-in) for co-localization correlation (Pearson correlation coefficient *r*). Bar graphs represent *r* values of three independent images selected from each group (mean ± SD) (lower panel).

Although little is known about the interplay between SUMO pathway and autophagy–lysosome system, it has been recently revealed that these two systems may cooperate in the regulated proteolysis. SUMO E3 ligase PIASγ, together with the lysine acetyltransferase Tip60, targets p53 for SUMO conjugation and the SUMOylated p53, in turn, activates autophagy (Naidu et al. 2012). It addition, SUMO-1 overexpression stimulates autophagosome formation and increases amyloid-β (Aβ) production via autophagy (Cho et al. 2015). Since autophagy can mediate the clearance of polyQ-expanded ataxin-3 (Menzies et al. 2010), we presumed that the over-expression of ataxin-3 alone is sufficient to induce autophagy. Indeed, LC3 puncta formation (autophagy marker) was observed even in cells expressing ataxin-3 only (Figure 3). Co-localization of ataxin-3 and LC3 signals, however, was not substantial. By contrast, SUMO-1 coexpression significantly enhanced the colocalization of LC3 and ataxin-3 signals (highlighted by arrows) (Figure 3, upper panels). These results were quantified by analysis of pixel intensity correlation. When SUMO-1 was co-expressed, the correlation coefficients for signals were very high (0.8–0.9) whereas the coefficients were below 0.7 in the absence of SUMO co-expression (Figure 3, lower panels). Above results raised a possibility that SUMO-1 promotes the degradation of ataxin-3 by facilitating the recruitment of SUMOylated substrates to autophagy rather than the induction of autophagy.

### A putative SIM and the degradation of ataxin-3

**Figure 4.**
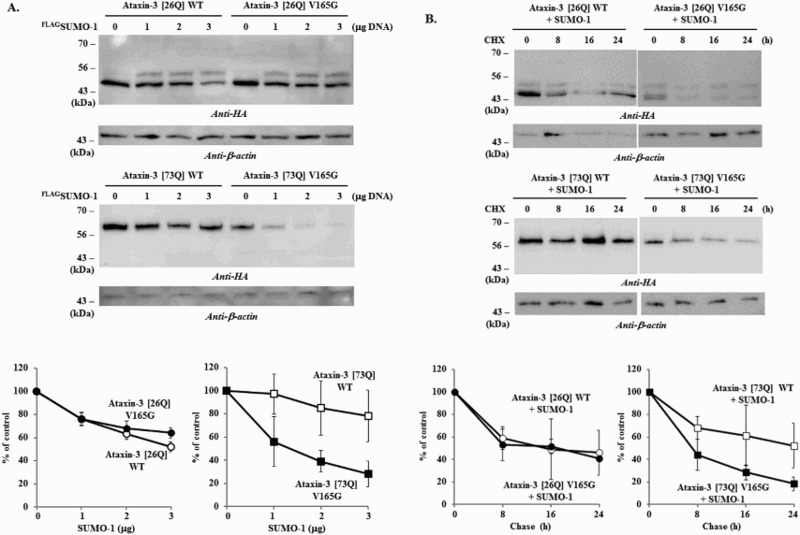
Effects of SIM mutation on the degradation of ataxin-3. To determine the role of SIM in ataxin-3 degradation, the effects of SUMO-1 overexpression on the normal (WT) and SIM mutant (V165G) of ataxin-3 (26Q and 73Q) were compared. **(**A**)** BOSC cells coexpressing HA-tagged ataxin-3 (1 µg) with different amount of FLAG-tagged SUMO-1 (0, 1, 2, 3 µg) were collected and the cell lysate was prepared. **(**B) After 24 h of transfection with ataxin-3 (normal and SIM mutant) and SUMO-1 (each 1 µg), cells were treated with 20 µg/ml of cycloheximide to block the synthesis of proteins. From this point (*t* = 0), cells were collected every 8 h (up to 24 h) and then processed for the immunoblot analysis. Immunoblot analysis of ataxin-3 with anti-HA antibody was performed as described in Figure 1. Graphs in the lower panels are the results of image quantification from three independent experiments. Bars = SD

Previously, we showed that a putative SIM (^162^IFVV^165^) is located adjacent to the SUMOylation site K166 (^165^VKGD^168^) in ataxin-3 (Jung & Lee 2013), which is analogous to SIM 2 (V/I-X-V/I-V/I) (Hecker et al. 2006). Analysis with GPS-SUMO program also confirmed that this motif is a potential SUMO-interaction site (Zhao et al. 2014; http://sumosp.biocuckoo.org). SIM can stimulate the interaction between SUMO moiety and STUbL and thereby facilitate the protein degradation (Maroui et al. 2012). We hypothesized that this putative SIM similarly affects ataxin-3 degradation and, to test this, constructed a SIM mutant. While the mutation of SIM (V165G) had no clear effect on the degradation of normal ataxin-3 [26Q], it distinctly enhanced the degradation of polyQ-expanded ataxin-3 [73Q] (Figure 4(A)). The chase experiments also showed similar results that V165G substitution facilitated the degradation of polyQ-expanded ataxin-3 [73Q] (Figure 4(B)). A possibility is that this particular substitution increases the interaction between SIM and proteolytic machinery rather than blocking the association. Although it remains to be determined why this particular substitution facilitated ataxin-3 degradation, these results indicate that this putative SIM influences the degradation by modulating the interaction between the substrates and proteolytic machinery.

## Discussion

While ubiquitylated proteins are primarily destined for destruction by proteasomes, SUMOylation of proteins is often associated with nuclear events including transcriptional regulation. SUMOylation modulates the functions of target proteins by affecting protein–protein interaction and subcellular localization; however, accumulating evidence suggests that SUMO also plays a role in proteolysis. Since Ub and SUMO both can modify the same acceptor lysine residues, the balance between two modifications may determine the stability of target proteins. In fact, global analysis of endogenous proteins revealed that almost a quarter of SUMOylated lysines are also ubiquitylated (Hendriks et al. 2014). Contrary to this competition model, the identification of specific E3 ligases recognizing protein-conjugated SUMO moieties (STUbL) and forming SUMO-Ub hybrid chains on substrates provides evidence for a cooperative action of Ub and Ub-like modifiers in protein degradation (Sriramachandran & Dohmen 2014; Liebelt & Vertegaal 2016).

Observations that numerous neurodegenerative proteins are modified by SUMO proteins indicated a similar role of SUMOylation in the proteostasis of disease proteins (Dorval & Fraser 2007; Liebelt & Vertegaal 2016). However, the effects of SUMOylation on stability, solubility and aggregation vary considerably depending on the context of target proteins. Furthermore, SUMO isoforms can exert distinct effects and conflicting results were often obtained. For example, knockdown of SUMO-1 and SUMO-2 did not change Aβ production whereas overexpression of SUMO-3 affects its production (Liebelt & Vertegaal 2016). There is also a discrepancy in the effects of SUMO-1 on ataxin-3. The mutation of SUMO acceptor lysine (K166) resulted in the reduced half-life of ataxin-3 although the effects were not clear (Zhou et al. 2013). On the contrary, we demonstrated that SUMO-1 overexpression facilitates degradation of ataxin-3 (Jung & Lee 2013). Perhaps different experimental approaches (mutation of acceptor lysines vs. SUMO overexpression) may account for such a discrepancy. Alternatively, SUMO isoforms may compete for the same acceptor lysine (K166) in ataxin-3 and SUMO-1 overexpression negatively affects the modification by SUMO-2/3. Indeed, SUMO-2/3-mediated ubiquitylation and degradation of ataxin-1 can be impeded by overexpression of SUMO-1 (Guo et al. 2014). In the following studies, it needs to be examined whether ataxin-3 is modified by SUMO-2/3 and if SUMO-1 negatively affects the modification by SUMO-2/3.

Initially, we expected that ataxin-3 degradation would be reduced by the SIM mutation since this motif likely plays a role in the interaction between ataxin-3 and proteolytic machinery. Surprisingly, we obtained the opposite results that V165G substitution facilitated the degradation of ataxin-3 [73Q]. Interestingly, several other studies employed a more dramatic substitution (e.g. VVDL-to-AAAA substitution for dengue virus NS5 protein or ILGV-to-KKGV substitution for human TRIM5α protein) to determine the functional requirement of SIM (Arriagada et al. 2011; Su et al. 2016). Perhaps the mild substitution we employed (Val to Gly) unexpectedly causes a prolonged association of ataxin-3 and the proteolytic machinery especially for polyQ-expanded ataxin-3 [73Q]. In the future studies, different substitutions should be designed and tested to confirm the requirement of SIM for SUMO-induced degradation of ataxin-3. Nevertheless, these results still support our notion that this putative SIM plays a role for proteolysis of ataxin-3. Several proteins functionally linked to UPS (e.g. S5a and Usp25) contain tandem SUMO- and Ub-interacting motifs (tSIM–UIM), which are considered as receptors recognizing SUMO–Ub hybrid chain (Guzzo & Matunis 2013). Ataxin-3 indeed contains multiple UIM domains downstream of SIM (Guzzo & Matunis 2013; Jung & Lee 2013). It would be interesting to test if SIM alone, or together with UIM, affects the degradation of ataxin-3, which could implicate the involvement of STUbL in this process. However, the findings that MG132 only partially stabilized ataxin-3 and that 3-MA prominently increased the steady-state level of ataxin-3 suggest a more active role of autophagy in SUMO-induced degradation.

As with other misfolded proteins prone to aggregate, the degradation of ataxin-3 (especially polyQ-expanded form) is promoted by autophagy inducers such as mTOR (mammalian target of rapamycin) inhibitors (Menzies et al. 2010; Ou et al. 2016). Although several attempts to pharmacologically induce autophagy and reduce aggregation formation of polyQ-expanded ataxin-3 have been reported, the underlying mechanism(s) is yet to be explained. Similar to other cellular pathways, the components of autophagy are also subjected to post-translational modifications (Wani et al. 2015). Recently, it became apparent that SUMO pathway and autophagy can cooperate in proteolysis. SUMOylation of components integral to autophagy (e.g. Vps34) is critical for the formation of autophagosomes (Yang et al. 2013). Ubc9, the E2 enzyme of SUMO pathway, increases autophagic flux and reduces aggregate formation in cardiomyocytes (Gupta et al. 2016). In addition, SUMOylation and acetylation play a crucial role in the regulation of p53-induced autophagy (Naidu et al. 2012). Presumably SUMO-1 overexpression leads to the enhanced SUMOylation of key regulatory components involved in autophagy and, as a result, the targets are then degraded by autophagy. On the other hand, our findings that SUMO overexpression did not increase LC3 puncta formation and instead enhanced the co-localization of ataxin-3 with LC3 raise a possibility that SUMO-1 overexpression increases SUMOylation of certain components involved in substrate (cargo) recruitment for autophagy, which in turn accelerates the turnover of substrates. However, it also needs to be examined whether SUMO-1 overexpression induces autophagy in various types of cells. While we failed to observe SUMO-induced activation of autophagy in BOSC cells (non-neuronal cells), the strong induction of autophagy in neuroglioma cells stably expressing SUMO-1 was reported (Cho et al. 2015). Maybe the extent of SUMO-induced autophagy activation is dependent on the types of cells employed. Further studies using reporter constructs (e.g. GFP-LC3) or different types of autophagy inhibitor (e.g. bafilomycin-A) could be helpful to understand if induction of autophagy by SUMO-1 overexpression is cell type-specific.

In conclusion, we present evidence that SUMO-1 induces ataxin-3 degradation via autophagy although UPS also contribute to this process to a certain extent. We also suggest that SUMOylation is possibly involved in the substrate recruitment by autophagy since SIM in ataxin-3 can influence the degradation of ataxin-3. Our results may provide an important clue for future attempts to study the detailed mechanism(s) by which the degradation of this disease-associated polyQ protein is regulated.
